# Supplementary material for: Unraveling the Complexity of the Dzyaloshinskii–Moriya Interaction in Layered Magnets: The Full Magnitude and Chirality Control
Source: Adv Mater. 2025 Jul 1;37(35):2500152. doi: 10.1002/adma.202500152 (PMC12412010; doi:10.1002/adma.202500152)
Supplement: Supplementary file 1 — Supporting Information [file ADMA-37-2500152-s001.pdf]

ADVANCED MATERIALS

Supporting Information

for *Adv. Mater.*, DOI 10.1002/adma.202500152

Unraveling the Complexity of the Dzyaloshinskii–Moriya Interaction in Layered Magnets: The Full Magnitude and Chirality Control

Khalil Zakeri, Albrecht von Faber, Sergiy Mankovsky and Hubert Ebert*

Supporting Information for

Unraveling the Complexity of the Dzyaloshinskii-Moriya Interaction in Layered Magnets: The Full Magnitude and Chirality Control

Khalil Zakeri,[1,*] Albrecht von Faber,[1] Sergiy Mankovsky,[2] and Hubert Ebert[2]

[1] *Heisenberg Spin-dynamics Group, Physikalisches Institut, Karlsruhe Institute of Technology, Wolfgang-Gaede-Strasse 1, D-76131 Karlsruhe, Germany*

[2] *Department of Chemistry and Physical Chemistry, LMU Munich, Butenandtstrasse 11, D-81377 Munich, Germany*

This PDF file includes:

- **Supporting Figure S1:** The magnon dispersion relation of different systems.
- **Supporting Note S1:** Details of the spin-, orbital- and layer-resolved density of states
- **Supporting Figure S2:** The calculated spin-, orbital- and layer-resolved DOS for different structures
- **Supporting Figure S3:** The calculated layer-resolved atomistic DM vectors $\vec{D}_{ij}$ and the effective DMI $\mathcal{D}$ for the Co/Fe/Co/Ir(001) layered structure
- **Supporting Figure S4:** Typical LEED patterns and EDS profile of the studied samples

* khalil.zakeri@partner.kit.edu

Supporting Figure S1

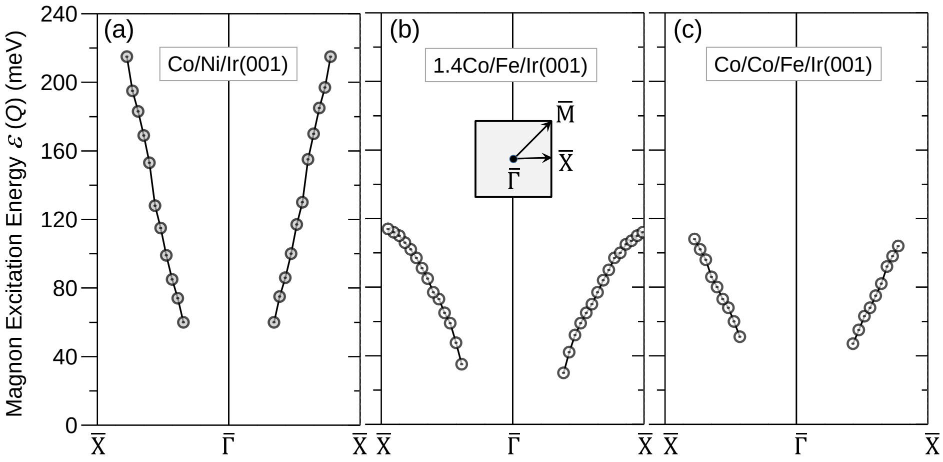

Supporting Figure S1. **The magnon dispersion relation of different systems.** The experimental results of Co/Ni/Ir(001), 1.4Co/Fe/Ir(001) and Co/Co/Fe/Ir(001) are shown in (a)–(c), respectively. The experimental data of Co/Co/Ir(001) may be found in Refs. [24, 51] of the manuscript. The surface Brillouin zone is shown in (b). The data for the negative values of Q were measured by reversing the sample magnetization $\vec{M}$ from the $[\bar{1}10]$- to the $[1\bar{1}0]$-direction, as explained in the main text of the manuscript.

Supporting Note S1. Details of the spin-, orbital- and layer-resolved density of states

The analysis of spin- and layer-resolved density of states (DOS), presented in Figure 5 of the main text, highlighted the critical features of DOS contributing to DMI. For the Co/Ni/Ir(001) system, a comparative analysis of the interfacial and bulk Ir atoms revealed a subtle modification exclusively in the Ir majority spin states. In particular, it was observed that the unoccupied majority states located slightly below an energy of about 1 eV are shifted towards the Fermi level. A detailed representation of the spin-, orbital- and layer-resolved DOS for the Co/Ni/Ir(001) system is provided in Supporting Figures S2(a)–(f). Detailed analysis reveals that these states primarily exhibit d_{yz}, d_{xz} and $d_{x^2-y^2}$ orbital character, as indicated by the gray, dark-blue and orange color, respectively, in Supporting Figure S2(a). These 5d states of Ir are responsible for the electronic hybridization. In order to disentangle the contributions of the in- and out-of-plane orbitals to this hybridization in Supporting Figure S2(d) we classify the d-orbitals into two categories: (i) d_{xy} and $d_{x^2-y^2}$ and (ii) d_{yz}, d_{xz} and d_{z^2}. A comparison between these two classes of d-orbitals is provided in Supporting Figures S2(d)–(f) for different layers. Supporting Figure S2(d) shows that both classes contribute to the hybridization. However, it seems that the contribution of the d_{yz}, d_{xz} and d_{z^2} orbitals is larger. This is also a result of the disparity in orbital hybridization, specifically three orbitals in the out-of-plane direction and two in the in-plane direction.

The energetic overlap of the Ir d-states, centered near 1 eV, with the electronic states of Ni is rather small, as can be seen from the data presented in Figure 5 of the main text. This is attributed to the fact that in Ni a large fraction of the electronic states are occupied and hence are located below the Fermi level. The limited number of states within this energy range are predominantly of d_{xz} and $d_{x^2-y^2}$ character. Again here both the in- and out-of-plane orbitals are involved, as demonstrated in Supporting Figure S2(e). The limited overlap between the Ni 3d and Ir 5d states results in a reduced atomistic DMI in Ni, consistent with the scenario that Hund's first rule drives the electronic hybridization, which dictates the DMI strength, as proposed in Ref. [41] of the manuscript. The partial DOS of the Co atoms shown in Supporting Figure S2(c) indicates that for this layer one would expect a moderate degree of hybridization due to the presence of enough Ir 5d states degenerated with the unoccupied minority states. These states are predominantly of d_{xz} d_{yz} and $d_{x^2-y^2}$ character, as can be seen from the data presented in Supporting Figure S2(c). Again both the in- and out-of-plane components contribute to the electronic hybridization and DMI, as demonstrated in Supporting Figure S2(f). The larger contribution of the out-of-plane orbitals is due to the larger number of orbitals of this kind. Note that due to the symmetry of the system the d_{xz} and d_{yz} orbitarls contribute equally to DOS.

The results of spin-, orbital- and layer-resolved DOS of Co/Fe/Ir(001) are summarized in Supporting Figures S2(i)–(l). The data should supplement those shown in Figures 5(e)–(g) of the main text. As it is observed in Figure 5(e) the shift in the Ir 5d states is remarkable. Particularly, the unoccupied minority states of the interface Ir atoms are shifted to higher energies, compared to the DOS of bulk Ir. This is a signature of a high degree of hybridization. These states are largely of d_{xz}, d_{yz} and $d_{x^2-y^2}$ nature, as can be concluded by analyzing the data shown in Supporting Figure S2(g). Notably, the Fe layer exhibits a remarkably high density of unoccupied states above the Fermi level [see Figure 5(f) of the main text and Supporting Figures S2(h) and S2(k)]. These states which primarily include states of d_{xy}, d_{xz}, d_{yz}, d_{z^2}, and partially states of $d_{x^2-y^2}$ character energetically degenerate with the Ir 5d states. The considerable amount of unoccupied d states of the surface Co layer above the Fermi level are mainly of d_{xz}, d_{yz} and $d_{x^2-y^2}$ character and both the in- and out-of-plane orbitals are involved, as can be concluded by looking at the data presented in Supporting Figures S2(i) and S2(l). All these observations explain the large values of atomistic DMI seen in the experiment.

If an additional Co layer is added to the structure one observes a considerable number of unoccupied minority states centered near 0.8 eV in DOS of the middle Co layer. These states are predominantly of $d_{x^2-y^2}$, d_{xy} character with contributions from d_{z^2} orbitals, as shown in Supporting Figures S2(o) and S2(s). They are located at the same energy as the d_{z^2} of the Fe layer, indicating a rather strong coupling between the middle Co layer and the Fe layer. On the other hand d_{xy} , d_{xz} d_{yz} and $d_{x^2-y^2}$ orbitals of the surface Co layer exhibit a considerable number of states near this energy, as seen in Supporting Figures S2(p) and S2(t). This is an indication of a strong hybridization between the two Co layers, leading to a spin-redistribution towards the surface. Such a spin-redistribution can explain the reduced interlayer DMI. A similar behavior has also been observed for the case of the interlayer Heisenberg exchange interaction as has been demonstrated in Ref. [43] of the main text.

Supporting Figure S2

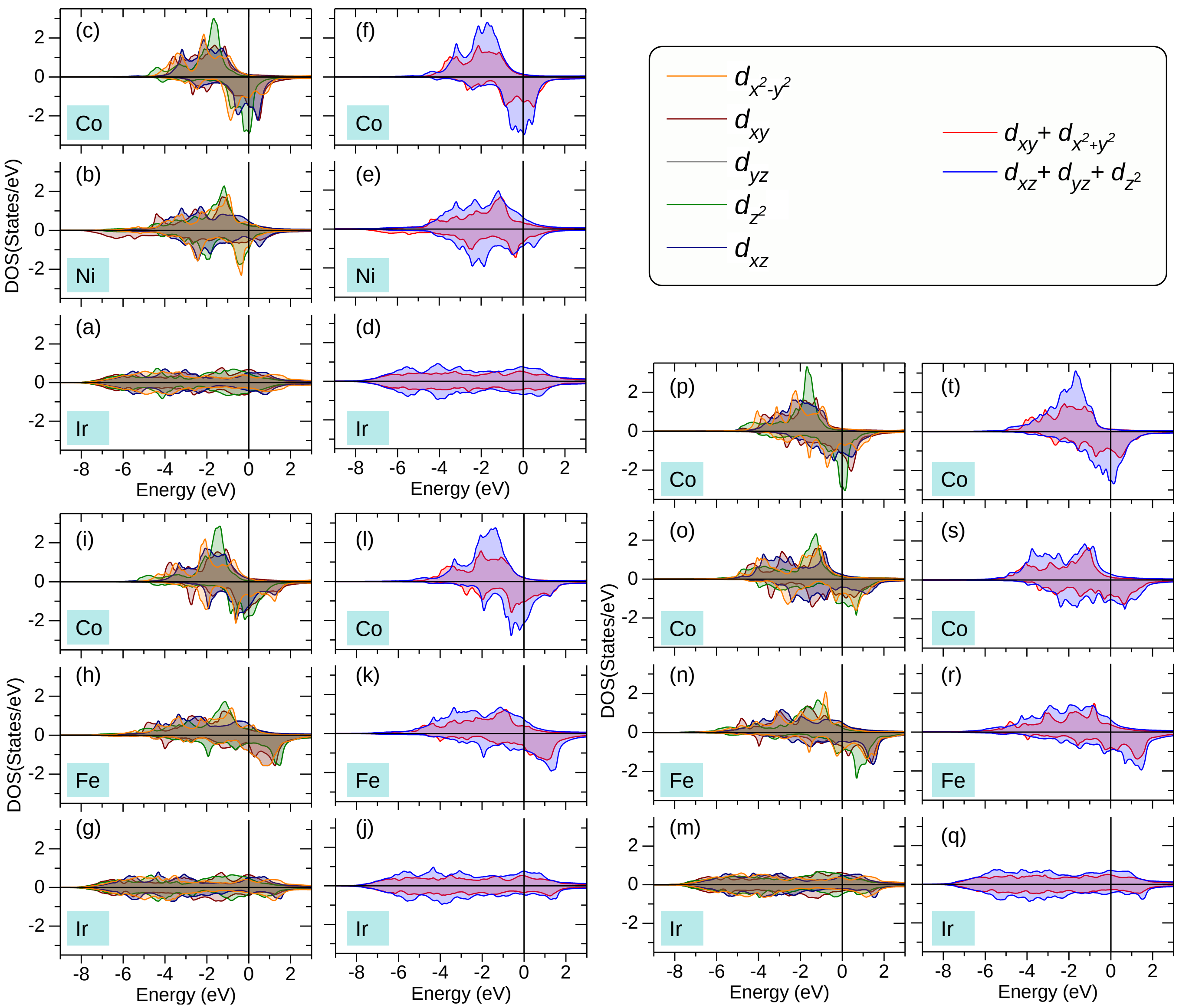

Supporting Figure S2. **The calculated spin-, orbital- and layer-resolved DOS for different structures.** The results of the Co/Ni/Ir(001), Co/Fe/Ir(001) and Co/Co/Fe/Ir(001) systems are shown in (a)–(f), (g)–(l) and (m)–(t), respectively. The individual contributions of different orbitals to the d states in the case of Co/Ni/Ir(001) are shown in (a)-(c) for each layer, as indicated. The contributions of the d_{xy} and $d_{x^2-y^2}$ orbitals and those of the d_{yz}, d_{xz} and d_{z^2} orbitals to the total density of the d states are shown in (d)–(f). For the Co/Fe/Ir(001) system the individual contributions of different d orbitals to the density of d states are shown in (j)–(i). The contributions of the d_{xy} and $d_{x^2-y^2}$ orbitals and those of the d_{yz}, d_{xz} and d_{z^2} orbitals to the total density of the d states are shown in (j)–(l). Likewise, for the Co/Co/Fe/Ir(001) system all the individual contributions of different d-orbitals are shown in (m)–(p). The contributions of the d_{xy} and $d_{x^2-y^2}$ orbitals and those of the d_{yz}, d_{xz} and d_{z^2} orbitals to the total density of the d states are shown in (q)–(t). For the sake of clarity the partial DOS, presented in (a)–(c), (g)–(i) and (m)–(p), are scaled with a factor of 4. Similar to Figure 5 of the main text, negative DOS values represent the minority states, while positive DOS values represent the majority states.

Supporting Figure S3

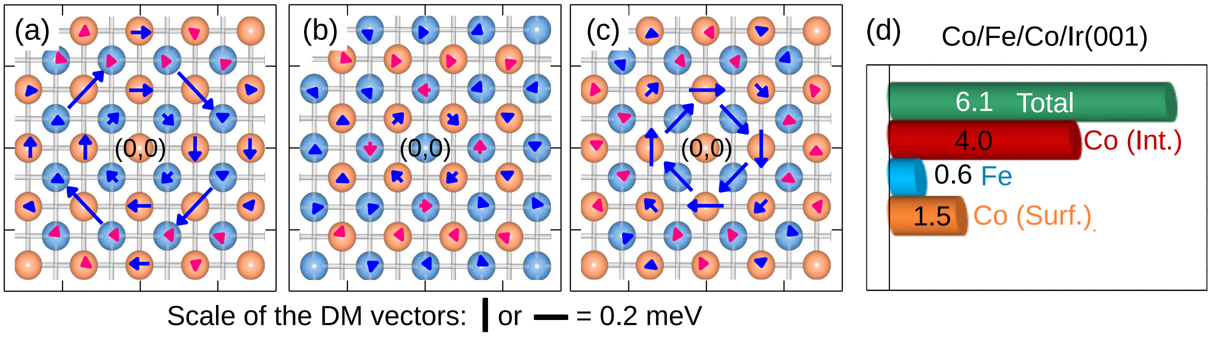

Supporting Figure S3. **The calculated layer-resolved atomistic DM vectors $\vec{D}_{ij}$ and the effective DMI $\mathcal{D}$ for the Co/Fe/Co/Ir(001) layered structure.** (a)–(c) The atomistic DM vectors within each atomic layer of the Co/Fe/Co/Ir(001) layered structure. The results for the case in which the origin site is located within the interface Co layer, the Fe layer and the surface Co layer are shown in (a), (b) and (c), respectively. For the sake of simplicity only the interaction between the neighboring layers are shown. The color of $\vec{D}_{ij}$s indicates their chirality (red for counter-clockwise and blue for clockwise). (d) The layer-resolved $\mathcal{D}$ given in meVÅ/μ_B.

Supporting Figure S4

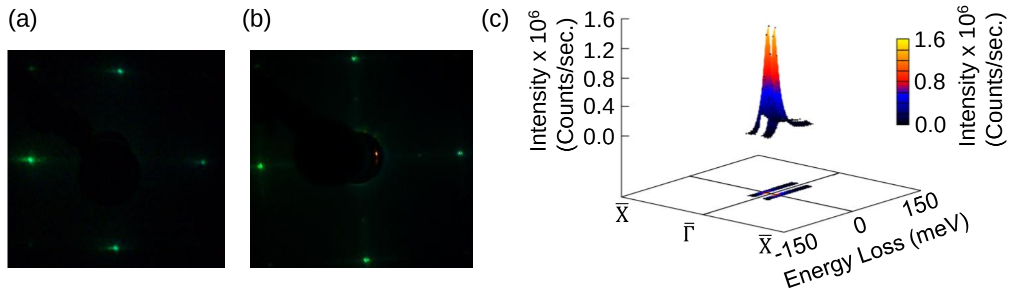

Supporting Figure S4. **Typical LEED patterns and EDS profile of the studied samples.** The low-energy electron diffraction (LEED) patterns of (a) Co/Fe/Ir(100) and (b) Co/Co/Fe/Ir(001), taken at electron energies of 99.7 and 99.3 eV, respectively. (c) The profile of the elastic diffuse scattering (EDS) recorded at an incident energy of 10 eV on the Co/Ni/Ir(001) sample. It shows the intensity distribution of the elasic diffuse scattering in the energy–momentum space.
